# Efficacy of Preoperative Physiotherapy Protocols in a 30-Year-Old Patient With Bilateral Osteoarthritis of Hip Secondary to Avascular Necrosis

**DOI:** 10.7759/cureus.46142

**Published:** 2023-09-28

**Authors:** Pooja R Tiwari, Pooja Dhage

**Affiliations:** 1 Department of Musculoskeletal Physiotherapy, Ravi Nair Physiotherapy College, Datta Meghe Institute of Higher Education and Research (Deemed to be University), Wardha, IND

**Keywords:** plyometrics exercises, proprioception training, physiotherapy, avascular necrosis, osteoarthritis of the hip

## Abstract

Osteoarthritis (OA) of the hip is a rare condition that occurs in adults can be a result of avascular necrosis or a history of steroids that can wear away the articulating cartilage of the hip joint causing friction, pain in the groin region, stiffness, and decreased functional mobility. We present a 30-year-old adult who came with chief complaints of pain in the groin region, stiffness, difficulty in walking, and experiencing pain while walking, which had reduced his activity of daily living. The investigation was done, and the patient was diagnosed with bilateral hip OA secondary to avascular necrosis. To reduce morbidity, preoperative physiotherapy management for eight weeks was planned and started before the operation. The purpose was to educate the patient about the condition, reduce pain, increase the ranges of the hip, improve strength, and provide gait re-education. We added basic proprioception training and plyometric exercises for the hip to improve strength and balance. At the end of the session patient, positive results were achieved. The progress of proprioception or balance training can be improved by using single-leg balance as an outcome measure. Hence, our study aims to use exercise therapy to reduce or postpone the need for hip arthroscopy. However, future research should focus on plyometric exercises for the lower limbs or any abnormalities associated with the lower limbs. However, they should be carried out when some recovery is observed in patients.

## Introduction

Osteoarthritis (OA) and avascular necrosis are different health conditions that affect the larger joints of the body. The disease rarely occurs in the 30s, especially in males. The hip is known as the enarthrosis (ball and socket) joint articulated with hyaline cartilage and helps in weight bearing. The joint manifests all the static and dynamic forces while standing, walking, and running. OA of the hip is a condition characterized by the loss of structural integrity of the cartilage that lines the articular surfaces of the joint. This destructive process leads to swelling, softening, decreased shock absorption, erosion, and fracture of underlying bones. The related symptoms are pain, reduced strength, loss of muscle bulk, and inability to use the affected limb allowing abnormal forces [1]. India is the second largest country with a prevalence of 62.36 million, which is 22%-39% per year, and the prevalence of OA of the hip in young adults is about 5.5% [2].

Avascular necrosis (AVN) also known as osteonecrosis of bone majorly occurs after primary OA of the hip and is caused due to temporary or permanent loss of blood supply leading to the breaking of a bone. The prevalence in India for AVN is 10.8% in males and 1.3% in females between the ages of 26 and 40 [3]. Physical activities include standing, walking, and running; during these activities, experiencing pain is a common problem, and its elevation in certain degrees reduces physical activities and increases the rate of disabilities in young individuals. Pain in OA followed by avascular necrosis of the hip has negative impacts on the lower back and knees, which alters the gait pattern in the individual. The abnormal gait or uneven gait patterns show inappropriate use of muscles in the lower extremities, often resulting in changes in gait such as irregularity, variabilities, and reduced pace.

To improve pain in individuals, it would be essential to understand the effect of gait patterns on pain by the therapist [4]. Physiotherapy always plays a crucial role in pain, deconditioning of muscles, functional disability, and reduced work capacity before and after the operation. It can be achieved by strengthening exercises for hip abductors as these are the main muscles affected by OA of the hip; osteo-rehabilitation improves pain and all outcomes [5]. In all phases of rehabilitation, balance and proprioception training are effective if they are structured specifically for balance enhancement, joint awareness, and consistent training volume [6]. The stretch-shortening cycle is commonly known as plyometric exercises and consists of repetitive jumping, hopping, bounding, and skipping movements [7].

## Case presentation

**Figure 1:**
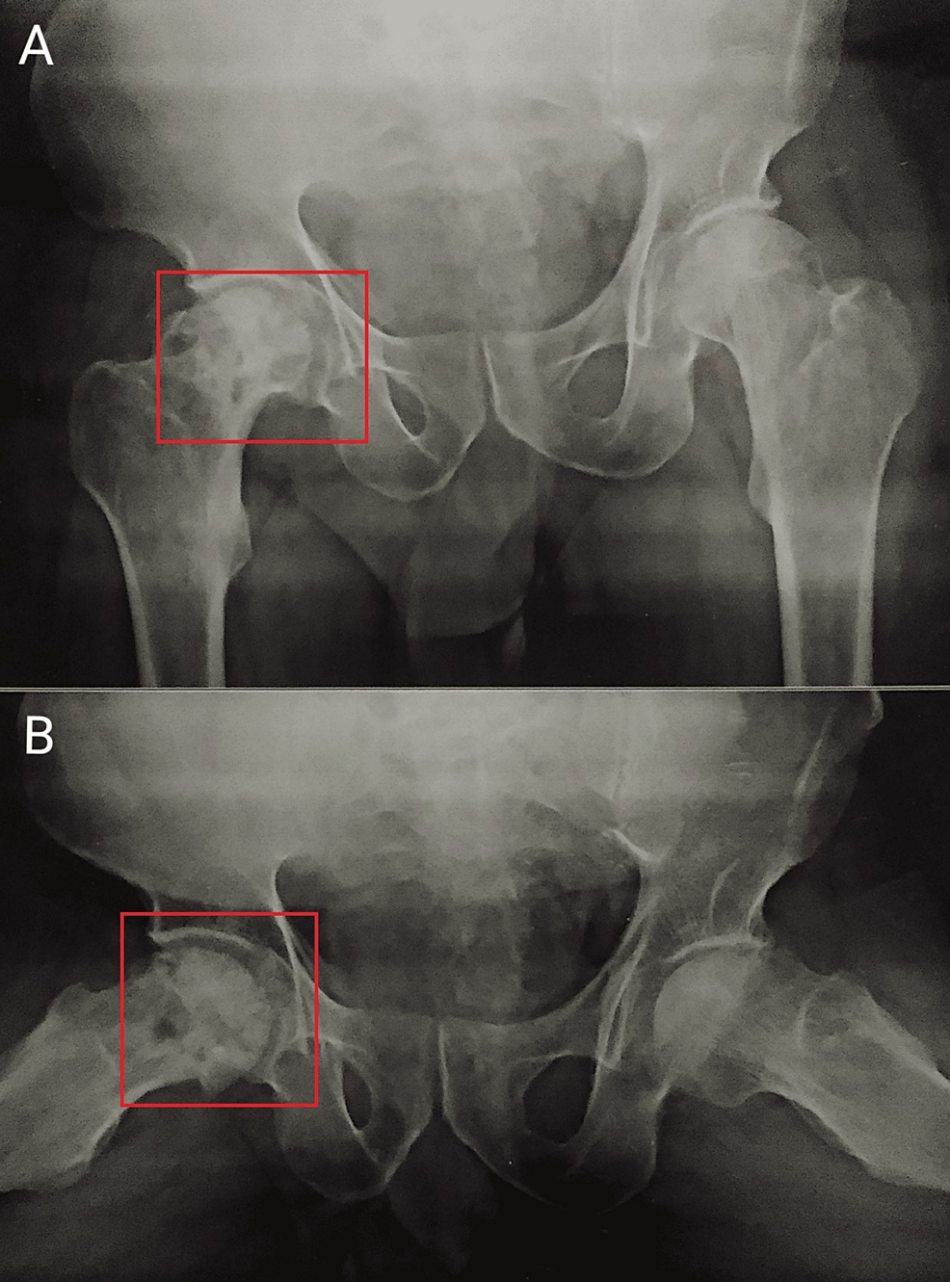
An X-ray from the anterior view of pelvis and hip joints. (A) and (B): Both the boxes on the right hip joint show reduced joint space.

**Figure 2:**
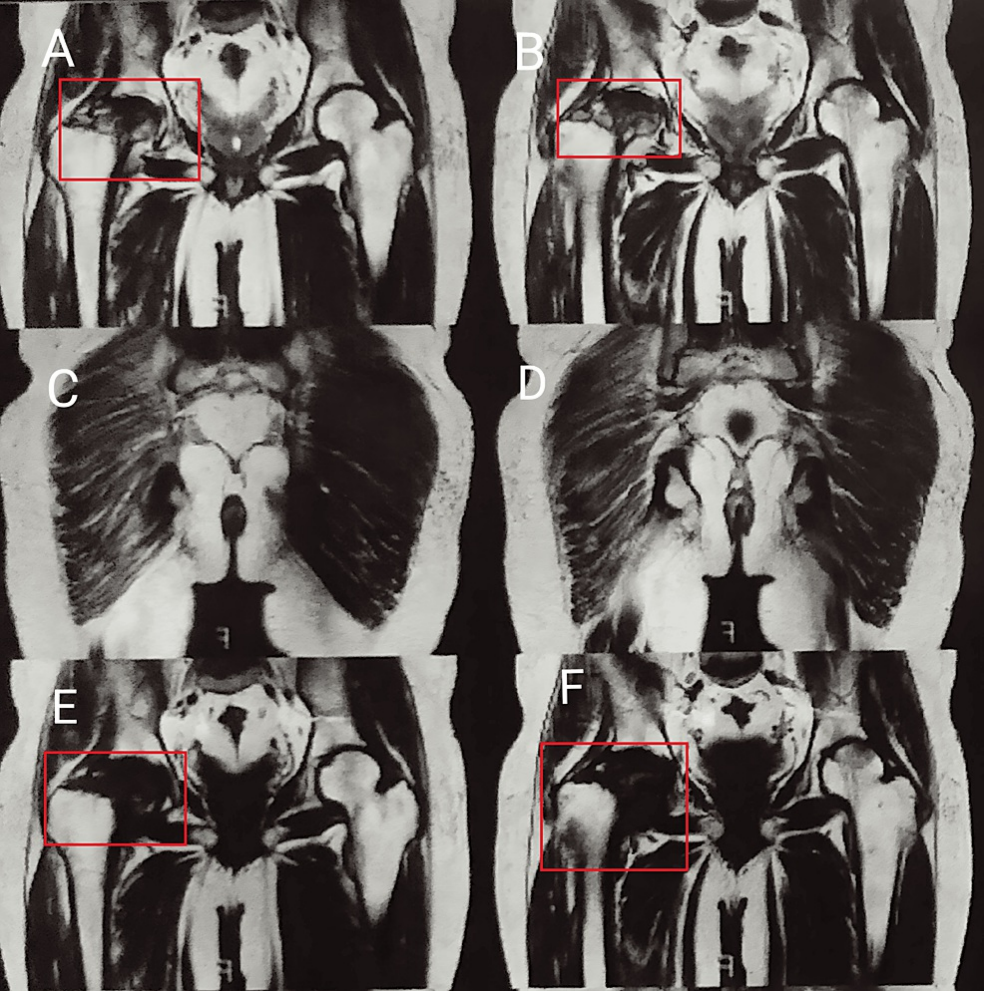
(A-F) MRI of the hip joint in which the boxes on images A, B, E, and F indicate avascular necrosis of the hip (Grade III)

A 30-year-old male patient, a driver by occupation, with right-hand dominance and no history of trauma, who was conscious and well oriented to time, place, and person came to the orthopedic department in the Acharya Vinoba Bhave Rural Hospital (AVBRH) hospital with chief complaints of pain around the groin and lower back region and joint stiffness of the hip. The patient explained a dull pain with a gradual onset that was progressive and aggravated more on limb movements and relieved by taking rest and medication; the pain on the numeric pain rating scale (NPRS) was 7/10 after the assessment. The range of motion of the hip was restricted, which made walking difficult. The patient had a previous history of hypothyroidism for three years, post-COVID, a history of steroids, and no previous surgical history. The patient had no significant family history. The patient was taking 100 mg aceclofenac for pain, which was prescribed by a local doctor. The patient was suggested for X-ray and MRI by orthopedic surgeons and was diagnosed with bilateral OA of the hip and avascular necrosis (right ˃ left) as shown in Figures 1, 2. The patient was advised for a hip arthroplasty operation and also referred to physiotherapy.

Clinical findings

**Figure 3:**
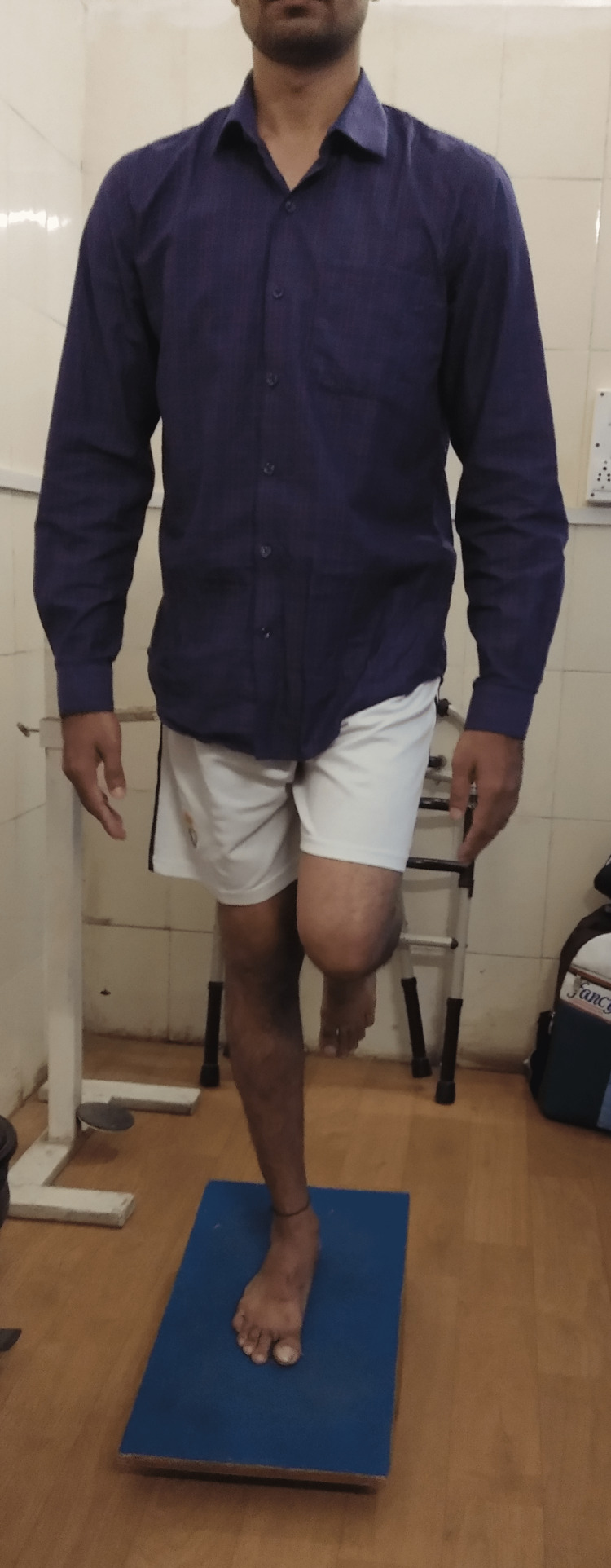
The proprioception training on the wobble board

The patient gave his consent for examination, and all the procedures for examination and further management were explained to him properly; then, we started the general examination. The patient’s height and weight were 1.55 meters and 55 kg, so the body mass index (BMI) was 22.60 kg/m^2^ according to which the build was moderate. The patient was hemodynamically stable and afebrile, with a pulse of 72 beats/min, respiratory rate of 12 breaths/min, and blood pressure of 120/80 mmHg. On observation, the attitude of the right lower limb from the hip was extended in supine lying and externally rotated with knee extension; the right anterior superior iliac spine (ASIS) appears at a higher level than the left, and a waddling gait was seen. On palpation, the temperature of the local area was increased, and tenderness was present over the anterior joint line. About 1 cm of true limb shortening was present on the right side. In this case, physiotherapy management was planned with appropriate goals and was followed for eight weeks as shown in Table 1. Figure 3 shows proprioceptive training on a wobble board. 

**Table 1:** Preoperative physiotherapy protocols

Goal	Rehabilitation	Time	Day/Week
To educate patient	We educated about the condition, the major risk factors, and dos and don’ts for example. Prevent overloading on joints.		Day 1
To reduce pain	Cryotherapy – ice packs given in resting position. Interferential current therapy (IFT) was suitable for pain-relieving. Controlled isometrics was done repeatedly, and they were not strong enough like what caused pain. We focused on pelvic muscles, hip abductors, and extensors with knee flexors and extensors.	10-15 minutes thrice a day;10 minutes once a day; 10 reps of 2 sets with 5-second holds	Week 1
To improve range of motion (ROM)	Relaxed active limb movements; small-range (close-kinematic-chain) stationary bicycle; proprioception neuromuscular facilitation (PNF) using hold-relax and contract-relax techniques.	10 reps for 30 seconds; 30 seconds to 1 minute of 2 sets once a day; once a day by therapist	Week 2
To improve the strength of muscles	Progressive resisted exercises were performed manually or using weight cuffs because isometrics was necessary to progress in graded steps. Plyometrics exercises with proper positioning of high knees, squat jumps, and jumping lunges.	5 minutes every hour	Week 2-3
To re-educate gait	Proper weight transfer during walking; properly wedged lateral insoles help relieve compressive forces on the knee; tandem walking, sideways walking, and stair climbing.		Week 4-5
To improve endurance	Aerobic or brisk walking [8] according to frequency, intensity, time, and type (FITT) principle: frequency - five times a week; intensity - easy to moderate or about 60%-70% of patient's maximum heart rate; time – 30-45 minutes per day; type-walking.	30-45 minutes a day	Week 6-7
To improve activities of daily living and home exercise program	Guidance for postures and activities causing excessive compression on joints; activities of daily living (ADLs) training.		Week 8

The range of motion and manual muscle testing are given in the form of outcome measures before and after physiotherapy in Table 2, and other outcome measures are shown in Table 3.

**Table 2:** Range of motion and manual muscle testing taken as outcome measures

Outcome measures	1st day of physiotherapy	1st day of physiotherapy	After 8 weeks of physiotherapy	After 8 weeks of physiotherapy
Range of motion	Right lower limb	Left lower limb	Right lower limb	Left lower limb
Hip flexion	0-70^°^	0-70^°^	0-90^°^	0-90^°^
Extension	0-20^°^	0-20^°^	0-30^°^	0-30^°^
Abduction	0-20^°^	0-30^°^	0-40^°^	0-40^°^
Adduction	0-30^°^	0-30^°^	0-40^°^	0-40^°^
Internal rotation	0-5^°^	0-10^°^	0-20^°^	0-30^°^
External rotation	0-10^°^	0-20^°^	0-30^°^	0-30^°^
Knee flexion	0-80^°^	0-90^°^	0-100^°^	0-110^°^
Extension	80-5^°^	90-5^°^	100-0^°^	110-0^°^
Ankle - Dorsiflexion	0-15^°^	0-15^°^	0-20^°^	0-20^°^
Plantar flexion	0-40^°^	0-45^°^	0-45^°^	0-50^°^
Manual muscle testing	Right lower limb	Left lower limb	Right lower limb	Left lower limb
Hip flexors	3/5	3/5	4/5	4/5
Extensors	3/5	3/5	4/5	4/5
Abductors	3/5	3/5	4/5	4/5
Adductors	3/5	3/5	4/5	4/5
Internal rotators	3/5	3/5	4/5	4/5
External rotators	3/5	3/5	4/5	4/5
Knee flexors	3/5	3/5	4/5	4/5
Extensors	3/5	3/5	4/5	4/5

**Table 3:** Outcome measures

Outcome measures	1st day of physiotherapy	After 8 weeks of physiotherapy
Numeric pain rating scale (NPRS)	7/10	3/10
Hip disability and osteoarthritis outcome score survey (HOOS)	Symptom subscale score – 35/100; Pain subscale score – 52/100; Daily living subscale score – 39/100; Sports and recreation subscale score – 25/100; Quality of life subscale score – 37.5/100	Symptom subscale score – 51/100; Pain subscale score – 69/100; Daily living subscale – 53/100; Sports and recreation subscale score – 40/100; Quality of life subscale score – 60/100
Time up and go test	14 seconds	8 seconds
Single-leg stance time	Unable to stand on one leg and took 30 seconds to hold the position.	Able to stand on one leg and took less than 5 seconds to hold the position.

## Discussion

OA is also known as the "wear and tear" disorder of joints; pathological changes occur in bone, cartilage, synovium, ligament, muscle, and periarticular fat, resulting in joint dysfunction, pain, stiffness, and functional limitations. A Kellgren-Lawrence grading system is typically used to grade radiographs, with Grade 0 representing no pathology; Grade 1 showing slight osteophytes; Grade 2 showing osteophytes; Grade 3 showing definite joint space narrowing; and Grade 4 showing severe joint space narrowing [9]. The alteration of gait pattern is observed in hip OA. Usually, individuals adopt compensatory trunk movement while walking. Upon inspection of the patient, this can be neglected; therefore, a body-fixed sensor camera was used, which showed increased trunk range of motion (ROM) and decreased pelvic ROM in a frontal plane, leading to a limping gait. So, the use of a body-fixed sensor-based gait analysis system can be beneficial in assessment and further treatment [10].

Exercise therapy is prescribed for all OA patients as the study conducted in 2013 explained that patient education on 12 weeks of exercise therapy sessions reduces the need for or postpones hip arthroplasty [11]. Proprioception of joints is called joint position sense, which helps in maintaining balance and posture by performing coordinated movements of joints. Impairment in proprioception affects neuromuscular control and biomechanics of joints and initiates degenerative changes in joints [12]. Zeng et al. explained that preoperative home-based strength training with tai chi improves the balance issue in patients with OA of the hip [13]. Individuals with plyometric training showed positive effects on any of these outcome variables, at least when comparing pre- to post-intervention results. Plyometrics consistently produces greater results than non-exercising controls in muscle strength, jump performance, and physical performance outcomes.

Individuals can benefit from plyometric training as a viable and safe alternative to traditional strength training, especially when supervised training is designed to improve an individual's dynamic neuromuscular performance [7]. The time up and go (TUG) test was created for older people to assess the risk of falls, but now this test is also used in different musculoskeletal conditions. The test is helpful in the assessment of strength, transitions, and dynamic gait balance. It shows test-retest reliability along with validity as TUG ˃ 10 shows the risk of falls in people with OA hip [14]. Single-leg balance is another objective outcome measure, which is also used for management purposes. The differences in gait patterns are due to abnormal weight shifts followed by the common finding of a reduction in the center of pressure and a change in the center of mass. So, single-leg balance is an essential component of training routines for practitioners, and they serve as a valuable source of information for exercise prescriptions that are based on scientific research [15].

## Conclusions

The preoperative physiotherapy protocols focused on hip strength and balance issues while standing and walking. We added some new interventions such as plyometrics exercises for improving hip strength and proprioception training for joint sense and balance. The eight-week session was started with specific goals in mind such as managing pain, increasing hip ranges, improving hip strength, and reducing difficulty while walking. This session was also helpful in promoting the patient’s quality of life and creating awareness about exercise therapy and its importance before and after surgical management. Postponing or reducing the need for hip arthroplasty can be achieved through exercise therapy sessions. Future studies should focus more on plyometrics exercises on lower limbs or any abnormal lower limb conditions but should be carried out when some sort of recovery is observed in patients. Using single-leg balance as an outcome measure can be effective in further proprioception or balance training.
